# Krüppel-like factor 9 and histone deacetylase inhibitors synergistically induce cell death in glioblastoma stem-like cells

**DOI:** 10.1186/s12885-018-4874-8

**Published:** 2018-10-22

**Authors:** Brian Tung, Ding Ma, Shuyan Wang, Olutobi Oyinlade, John Laterra, Mingyao Ying, Sheng-Qing Lv, Shuang Wei, Shuli Xia

**Affiliations:** 1 Hugo W. Moser Research Institute at Kennedy Krieger, The Johns Hopkins School of Medicine, 707 N. Broadway, Room 400K, Baltimore, MD 21205 USA; 2 Department of Neurology, Johns Hopkins School of Medicine, Baltimore, MD USA; 3 Department of Oncology, Johns Hopkins School of Medicine, Baltimore, MD USA; 4 Department of Neuroscience, Johns Hopkins School of Medicine, Baltimore, MD USA; 5 Department of Neurosurgery, Xinqiao Hospital, Third Military Medical University, Chongqing, 400037 China; 6 Department of Respiratory and Critical Care Medicine, Tongji Hospital, Tongji Medical College Huazhong University of Science and Technology, Wuhan, 430030 China

**Keywords:** Glioblastoma, Glioblastoma stem cells, Cancer stem cells, KLF9, HDAC inhibitors, Apoptosis, Necropotosis

## Abstract

**Background:**

The dismal prognosis of patients with glioblastoma (GBM) is attributed to a rare subset of cancer stem cells that display characteristics of tumor initiation, growth, and resistance to aggressive treatment involving chemotherapy and concomitant radiation. Recent research on the substantial role of epigenetic mechanisms in the pathogenesis of cancers has prompted the investigation of the enzymatic modifications of histone proteins for therapeutic drug targeting. In this work, we have examined the function of Krüppel-like factor 9 (KLF9), a transcription factor, in chemotherapy sensitization to histone deacetylase inhibitors (HDAC inhibitors).

**Methods:**

Since GBM neurosphere cultures from patient-derived gliomas are enriched for GBM stem-like cells (GSCs) and form highly invasive and proliferative xenografts that recapitulate the features demonstrated in human patients diagnosed with GBM, we established inducible KLF9 expression systems in these GBM neurosphere cells and investigated cell death in the presence of epigenetic modulators such as histone deacetylase (HDAC) inhibitors.

**Results:**

We demonstrated that KLF9 expression combined with HDAC inhibitor panobinostat (LBH589) dramatically induced glioma stem cell death via both apoptosis and necroptosis in a synergistic manner. The combination of KLF9 expression and LBH589 treatment affected cell cycle by substantially decreasing the percentage of cells at S-phase. This phenomenon is further corroborated by the upregulation of cell cycle inhibitors p21 and p27. Further, we determined that KLF9 and LBH589 regulated the expression of pro- and anti- apoptotic proteins, suggesting a mechanism that involves the caspase-dependent apoptotic pathway. In addition, we demonstrated that apoptosis and necrosis inhibitors conferred minimal protective effects against cell death, while inhibitors of the necroptosis pathway significantly blocked cell death.

**Conclusions:**

Our findings suggest a detailed understanding of how KLF9 expression in cancer cells with epigenetic modulators like HDAC inhibitors may promote synergistic cell death through a mechanism involving both apoptosis and necroptosis that will benefit novel combinatory antitumor strategies to treat malignant brain tumors.

## Background

Glioblastoma (GBM), grade IV astrocytoma, is the most aggressive primary brain tumor in adults. Despite current advances in surgery, radiotherapy, and chemotherapy, GBM remains incurable and claims roughly 17,000 lives each year in America [1]. Like other solid tumors, GBMs are an accumulation of heterogeneous cell populations comprised of a select few cancer stem cells (CSCs) that are able to initiate and sustain tumor growth [2]. CSCs are multipotent, able to differentiate into multiple cell types to make up the tumor bulk [3], and display signature characteristics of self-renewal and unlimited growth potential. Due to upregulated multi-drug transporters, altered anti-apoptotic machinery, and enhanced DNA damage response, CSCs are relatively resistant to most chemotherapy and radiotherapy [4], therefore substantially contribute to tumor metastasis and recurrence. GBM stem-like cells (GSCs) grow in vitro as non-adherent clonal multicellular neurospheres and efficiently initiate tumor xenografts that recapitulate the genetic and histopathological features of the original neoplasm from which they were derived [5]. Therefore, targeting GSCs or their tumor-initiating capacity will provide mechanistic insights that may more efficaciously treat this deadly cancer.

Various approaches have been tested to induce GSC differentiation or cell death to reduce their tumor-initiating potential, such as treatment with bone morphogenic protein (BMP) [6], histone deacetylase inhibitors [7], retinoic acid [8], and overexpression of transcription factors [9]. The Krüppel-like factors (KLFs) consists of 17 evolutionarily conserved zinc finger transcription factors with diverse regulatory functions [10]. By binding to GC-GT rich regions in promoters/enhancers, KLFs regulate a variety of cellular functions such as proliferation, cell survival and differentiation [11, 12]. It has been reported that KLF family members act as tumor suppressors and/or oncogenes under distinct cellular context [13, 14]. Krüppel-like factor 9 (KLF9), also known as basic transcription element-binding protein 1 (BTE-B1), has been found downregulated in a number of cancers including endometrial carcinoma and colorectal cancer [15, 16]. Our research group previously showed that expression of KLF9 in GBM was low [9] and found it upregulated in response to diverse differentiation signals [7, 8]. Moreover, KLF9 induces GSC differentiation and inhibits GSC self-renewal and xenograft growth in vivo [9, 17].

DNA methylation and histone modifications are epigenetic mechanisms that contribute to the pathogenesis of cancer, including GBM [18]. Enzymatic modifications of histone proteins have being exploited for therapeutic cancer targeting. Histone deacetylase (HDAC) inhibitors consist of a group of agents that block histone de-acetylation and neutralize positively charged lysine residues on histone tails, thereby altering chromatin structure and gene transcription [19]. HDAC inhibitors have been reported to kill a variety of tumor cells through diverse mechanisms [20, 21], including disruption of co-repressor complexes, induction of oxidative injury, upregulation of death receptor and ligand expression, generation of lipid second messengers, interference with chaperone protein function, modulation of NFκB activity, mitotic catastrophe, and interference with DNA repair. Thus, HDAC inhibitors reduce tumor growth mainly by inducing cell growth arrest and cell death (i.e. apoptosis and autophagy), to a less extent by modulating tumor cell migration and tumor-microenvironment interactions [22]. Several HDAC inhibitors, such as vorinostat (SAHA) and panobinostat (LBH589), have been approved by the U.S. Food and Drug Administration (FDA) for the treatment of several malignancies. LBH589 is a non-selective histone deacetylase inhibitor that has been approved for the treatment of various cancers including multiple myeloma [23]. In our own laboratory, we have previously shown that HDAC inhibitors are potent differentiation agents in GSCs. In the current study, GSCs were used to examine the function of KLF9 in chemotherapy sensitization to HDAC inhibitors. We found that KLF9 induction synergizes with HDAC inhibitors to induce cell death in GSCs through a mechanism that involves both apoptosis and necroptosis.

## Methods

### Materials

Reagents were purchased from Sigma (St. Louis, MO) unless otherwise mentioned. Drugs were made in stock and diluted in cell culture medium. Doxycycline was dissolved in water. Stock solutions of LBH589 (Novartis, Basel, Switzerland) was dissolved in DMSO and added to the media at the indicated concentrations. Pan-caspase inhibitor Z-VAD-FMK was dissolved in DMSO at 50 mM.

### Primary GBM neurosphere culture

The human glioblastoma neurosphere lines HSR-GBM1A (0913) and HSR-GBM 1B (0627) were obtained from Vescovi and colleagues in 2006 and maintained in serum-free medium supplemented with epidermal growth factor (EGF) and fibroblast growth factor FGF [5, 24]. Both cells lines are free from mycoplasma and authenticated with short tandem repeat (STR) profiling by Johns Hopkins Genetic Resources Core facility using Promega GenePrint 10 system (Madison, WI). The use of these cell lines does not require ethics approval from Hopkins as they were from surgical waste. Cells were incubated in 5% CO_2_/95% air condition at 37 °C. The cells were plated at a density of 5000 live cells/cm^2^ for most studies.

### Western blot analysis

Total cellular protein was extracted with radioimmunoprecipitate assay buffer (RIPA) [25, 26] containing protease and phosphatase inhibitor mix (Calbiochem, Darmstadt, Germany). SDS-polyacrylamide gel electrophoresis (PAGE) was performed with 30 μg cellular protein per lane using 4–12% gradient Tris-glycine gels. Western Blot was performed using Quantitative Western Blot System (LI-Cor Biosciences, Lincoln, NE) in accordance to the manufacturer’s instructions. All primary antibodies used for Western blot were obtained from Cell Signaling Technology (Beverly, MA) unless otherwise stated and the concentrations used for Western blotting were according to the manufacturers’ recommendations. Secondary antibodies were the IRDye infrared dyes and protein levels were quantized with Odyssey Infrared Imaging System (LI-COR Biosciences) [27].

### MTS assay

Neurosphere proliferation was measured by MTS (3-(4,5-dimethylthiazol-2-yl)-5-(3-carboxymethoxyphenyl)-2-(4-sulfophenyl)-2H-tetrazolium) assay. Briefly, cells were plated in 24-well plates at a density of 10,000 cells/well in 1.0 ml volume of human neurospheres medium. Wells were set up in triplicate per condition using the drug concentrations: doxycycline (dox) (0.01 μg/mL to 1 μg/mL), Panobinostat (LBH-589, 25–200 nmol/L). Cellular enzymatic activity was determined by incubation with MTS for 4 h and quantified in a spectrometer at settings between 500 and 600 nm. The results are expressed as a percentage of absorbance measured in control cultures after subtracting the background absorbance from all values. We used the isobologram eq. [28] to determine the interaction of two reagents: I_x_ = (a/A) + (b/B). In this equation, A is the IC_50_ concentration of Dox; B is the IC_50_ concentration of LBH-589; a and b are the concentration of Dox (a) or LBH-589 (b) required to produce the same effect in combination with the other agent. If I_x_ < 1, the combination effect is synergistic; if I_x_ = 1, the combination effect is additive; If I_x_ > 1, the combination effect is antagonistic.

### Cell cycle analysis

Cell cycle analysis was performed according to Oyinlade et al. [29, 30]. Briefly, cells were seeded on 10cm^2^ dish at a density of 5000 cells/cm^2^ for 24 h followed by drug treatment for 24 h. To harvest, cells were trypsinized and dissociated by pipetting, fixed with 75% ethanol at 4 °C for 30 min. Cells were then incubated with DNase-free RNase at 37 °C for 30 min followed by incubation with propidium iodide (PI, 100 ng/ml) for 1 h at 37 °C before subjected to cell cycle analysis with flow cytometry. To synchronize the cells, thymidine (50 mmol/L) was added to cells for 18 h. Cells were then spun down and washed with phosphate buffered saline (PBS) and replated in normal GBM medium. Cells were treated with Dox and LBH589. Cells were left to incubate for 6–42 h prior to harvest for cell cycle analysis. Flow cytometry analyses were performed on a FACscan (Becton-Dickinson, Mountain View, CA). The percentage of cells at each cell-cycle phase (G1/G0, S and G2/M) was analyzed using CellQuest software (Becton-Dickinson).

### Flow cytometry / apoptosis assay

Apoptosis was quantified using the Annexin V-FITC / propidium iodide apoptosis kit (BD Biosciences, San Diego, CA) as previously reported [31]. Briefly, U87 cells were trypsinized, pelleted by centrifugation, resuspended in Annexin V binding buffer (150 mM NaCl, 18 mM CaCl_2_, 10 mM HEPES, 5 mM KCl, 1 mM MgCl_2_). FITC-conjugated Annexin V (1 μg/ml) and PI (50 μg/ml) were added to cells and incubated for 30 min at room temperature in the dark. Analyses were performed on a FACscan (Becton-Dickinson, Mountain View, CA). The data were analyzed with CellQuest software (Becton-Dickinson) [32].

### Statistical analysis

Statistical analysis was performed using Prism software (GraphPad, La Jolla, CA). Post hoc tests included Students T-test and Tukey multiple comparison tests as appropriate. All in vitro experiments reported here represent at least three independent replications. All data are represented as mean value ± standard error of mean (S.E.) from combined analysis of three independent experiments; significance was set at *P* < 0.05.

## Results

### KLF9 expression and HDAC inhibitor LBH589 potentiated GBM stem-like cell death

**Fig. 1:**
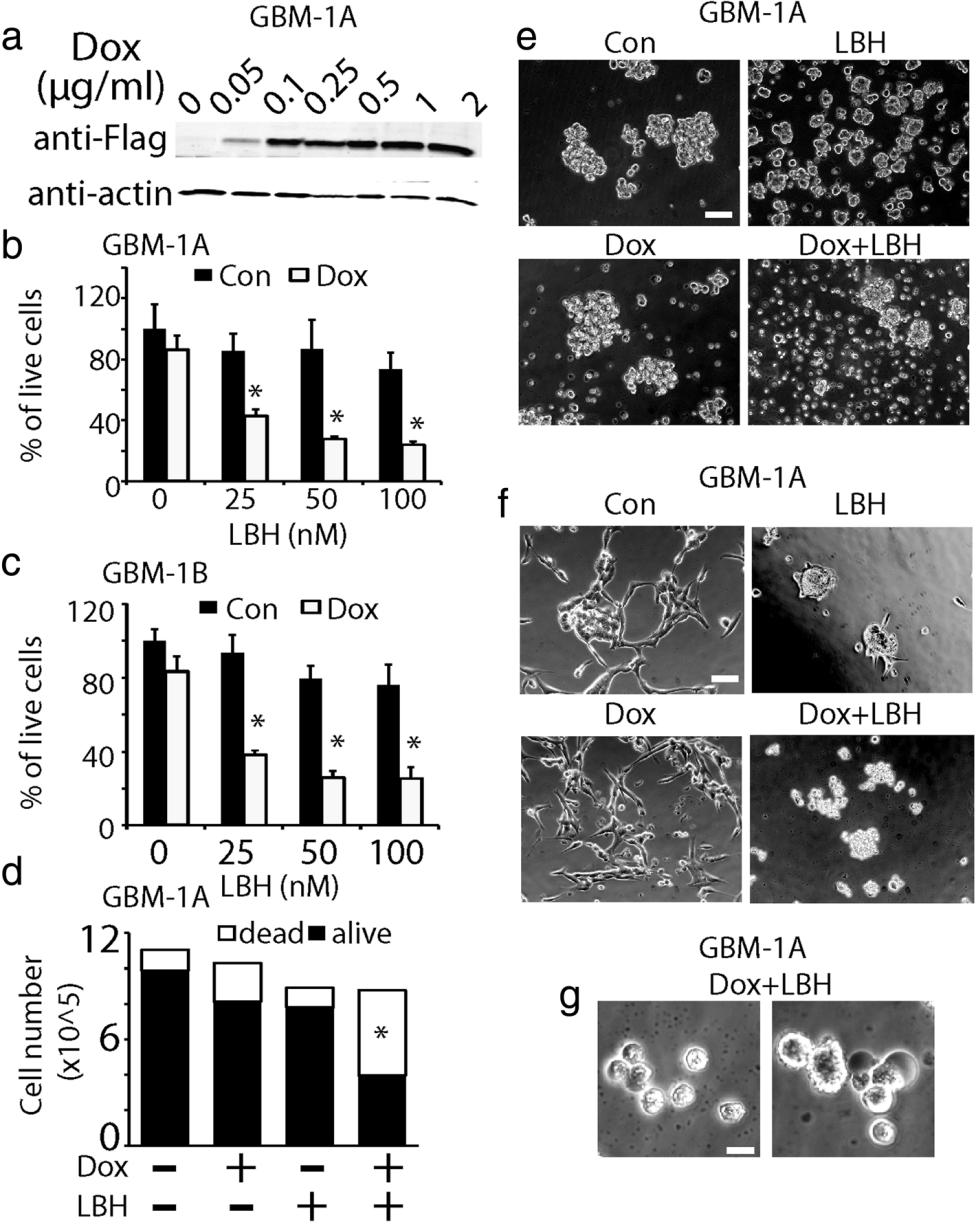
GBM stem cell (GSC) viability was reduced by forced KLF9 expression and HDAC inhibitor. **a** Dox-induced KLF9 expression in GBM1A neurosphere cells in a dose-dependent manner. **b** MTS assays of cell viability in GBM1A cells treated with Dox (0.1 μg/ml), LBH589 (25–100 nmol/L) alone, and combined treatment for 48 h. **c** Similar enhanced cell death in GBM1B cells was observed when treated with Dox (0.1 μg/ml) and LBH589 at 25–100 nmol/L. **d** Trypan blue staining showing that the combined treatment of Dox and LBH598 significantly induced cell death in GSCs. Neurosphere cells were dissociated to single cell suspension by vigorously pipetting and stained with trypan blue for 10 min. Number of trypan blue positive cells (white bar) and live cells (black bar) were counted and plotted. **e** Phase contrast photographs of GBM1A neurosphere cells treated with Dox, LBH589 alone, and combined treatment for 48 h. Upon combined Dox + LBH589 treatment, neurospheres displayed massive cell death. Bar = 50 μm. **f** GBM1B cells were grown on laminin-coated surfaces as adherent cultures and treated with Dox + LBH589. After 48 h, significant cell death was observed under the combined treatment. Bar = 50 μm. **g** A close look at the morphology of dead cells in the Dox + LBH treated cultures revealed the process of both apoptosis and necrosis. The non-viable cells exhibited apoptotic cell bodies (left panel) and membrane rapture, ghost-like cell debris (right panel), indicating a necrotic phenotype. Bar = 10 μm. *: *P* < 0.05

Two human GBM neurosphere cell lines HSR-GBM1A (GBM1A) and HSR-GBM1B (GBM1B) were used in the current study. The neurosphere cultures were enriched for GSCs, and have been extensively characterized by us and others in terms of their stem cell marker expression, differentiation potential, and tumor initiation capacity [8, 9, 33–35]. We engineered tet-on stable neurosphere cell lines to express flag-tagged KLF9. Upon doxycycline (Dox) addition, KLF9 expression was induced in a dose-dependent manner (0.05–2 μg/ml) as revealed by Western blot analysis (Fig. 1a). Dox at 0.1–1 μg/ml induced ~ 10–15 fold KLF9 expression, which was found to reduce stem cell marker expression and decrease tumor-propagating capacity of GSCs as we previously reported [9, 17]. In this study, we investigated the cell death responses of GSCs to KLF9 expression alone and in conjunction with other anti-tumor reagents.

We quantified GSC viability with 3-(4,5-dimethylthiazol-2-yl)-2,5-diphenyltetrazolium bromide (MTS) assays, which is based on NAD(P)H-dependent dehydrogenase enzymatic activities in live cells. Forced expression of KLF9 marginally decreased GBM1A neurosphere growth in vitro*,* as approximately 80% cells were viable after Dox (0.1 μg/ml) treatment for 48 h, indicating that KLF9 expression had minimal effect on cell proliferation and cell death (Fig. 1b). We then examined tumor cell death when forced KLF9 expression was combined with a variety of anti-tumor reagents, including chemotherapeutic drugs and epigenetic modulators. We tested temozolomide, camptothecin, and DNA methylation inhibitor 5-aza-2′-deoxycytidine. None of these drugs synergized with KLF9 to kill tumor cells as measured by MTS assays. However, the combination of KLF9 expression and HDAC inhibitor LBH589 dramatically induced GSC death. Compared to control, the administration of LBH589 alone, ranging from 25 to 100 nmol/L caused marginal cell number loss, with roughly 87% cells alive in GSC cultures treated with LBH589 at 25 nmol/L for 48 h. However, the combination of KLF9 induction and LBH589 dramatically decreased GSC viability. GBM1A cells simultaneously treated with Dox (0.1 μg/ml) + LBH589 (25 nmol/L) resulted in only 38% live cells after 48 h incubation, which was far less than the live cells from the additive effect of Dox and LBH589 (80% × 87% =70%) (*P* < 0.05, Fig. 1b). Similar cell number reduction induced by KLF9 expression + LBH589 was observed in GBM1B neurosphere cells (Fig. 1c). Because MTS assays only measures total live cells in cultures, which could be a result of adjuvant cell growth inhibition and cell death, the decreased cell number induced by KLF9 expression and LBH589 was further analyzed by trypan blue exclusion analysis. Under control conditions, the baseline trypan blue positive cells were ~ 11%, possibly due to mechanical damage of cells when dissociating neurospheres to single cells for counting. We found there was no significant increase in trypan blue positive cells in the presence of Dox (0.1 μg/ml, 18%) or LBH589 (25 nmol/L, 12%), indicating that KLF9 expression alone or LBH589 alone did not induce cell death. However, Dox + LBH589 dramatically increased the percentage of trypan blue positive cells to 55% (*P* < 0.05), confirming the combinatory effect of KLF9 induction and LBH589 on enhanced GSC death (Fig. 1d).

In addition, phase contrast microscopy demonstrated cell death induced by KLF9 expression and LBH589 in GSC cultures in both floating neurospheres (Fig. 1e) and adherent cells on laminin-coated surfaces (Fig. 1f), with observations of morphological changes under various treatments. Laminin by itself neither induced cell death nor affected the stem-like features of GSCs, as previously reported by others [36]. After 48 h of the combined treatment, the morphology of cell death showed a mixture of apoptosis and necrosis. Some dead cells exhibited apoptotic bodies and condensed nuclei (Fig. 1g, left panel). On the other hand, some non-viable cells portrayed features of necrotic cell death with swollen cell bodies, burst membrane, as well as ghost-like bodies (Fig. 1g, right panel).

Since Dox has been shown to cause mitochondrial toxicity [37], additional investigations were warranted to rule out the possibility that Dox itself was inherently toxic to GSCs*.* To validate that the cell death phenomenon we observed was due to KLF9 function instead of Dox itself, we treated parent GSCs with Dox + LBH589 and did not appreciate any significant cell death by MTS assays and cell counting (data not shown).

### Synergistic inhibition of GSC viability by KLF9 expression and HDAC inhibitors

**Fig. 2:**
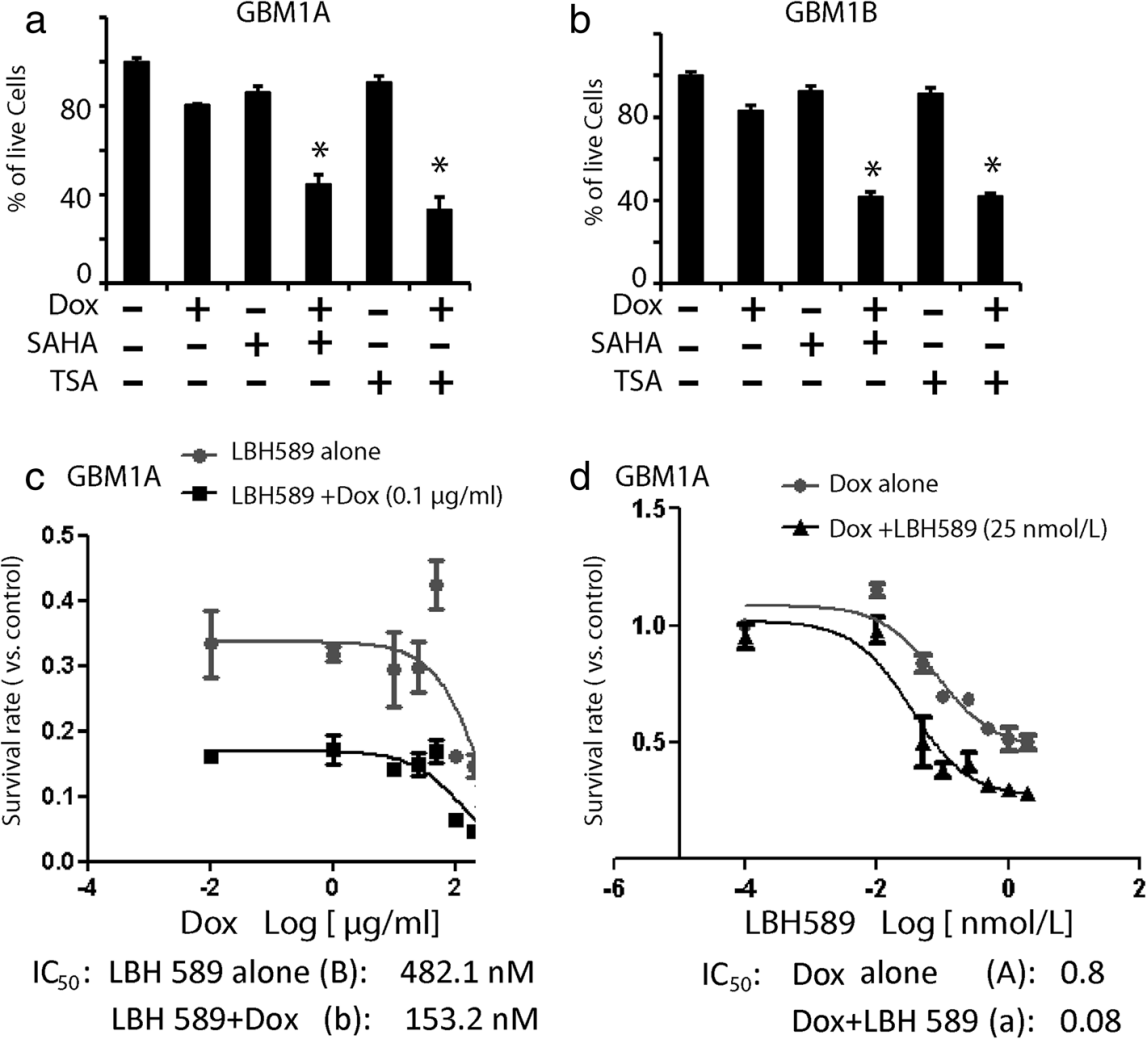
Isobologram analysis indicated KLF9 expression and HDAC inhibitors synergistically induced GSC death. **a**, **b** Enhanced cell viability loss induced by KLF9 expression and HDAC inhibitors SAHA and TSA in GBM1A (**a**) and GBM1B cells (**b**). MTS assays demonstrated that SAHA (10 μmol/L) or TSA (50 nmol/L) alone did not markedly induce cell death. The combination of Dox and SAHA or TSA induced ~ 60–65% GSC loss after 48 h of simultaneous treatment. **c** Systematic characterization of cell viability (control as 100%) in GSCs treated with LBH589 alone at different concentrations, or in the presence of Dox (0.1 μg/ml). IC_50_ was calculated by Graphpad Prism. **d** Cell viability assays (control as 100%) under the treatment of Dox at different concentrations, or in the presence of LBH589 (25 nmol/L). Isobologram index (Ix) was calculated as 0.41, indicating synergistic cell killing effect of these two treatments. *: *P* < 0.05

We further examined whether concurrent KLF9 expression alongside other HDAC inhibitors, i.e. vorinostat (SAHA) or trichostatin (TSA), enhanced cell death in GSCs. MTS assays indicated similar loss in cell viability in KLF9-expressing GSCs when treated with SAHA or TSA (Fig. 2a, b), suggesting a universal tumor cell killing effect of KLF9 in conjunction with HDAC inhibitors. In our following experiments, we mainly studied cellular responses to KLF9 expression in the presence of LBH589. Isobologram analysis [31, 38] determined KLF9 expression synergized with LBH589 to kill GSCs. We calculated the median inhibitory concentration (IC_50_), defined as the concentration of drug that induced 50% of cell number loss, of each agent alone and in the presence of one other.. In the absence of Dox, only high concentrations of LBH589 (> 500 nmol/L) induced cell number loss in GSCs (Fig. 2c). This was changed by co-application of a sub-lethal concentration of Dox (0.1 μg/ml) to induce KLF9 expression. Dox reduced the IC_50_ of LBH589 from 482 nmol/L to 153 nmol/L. On the other hand, adding LBH589 altered cellular response to Dox. LBH589 (25 nmol/L) together with Dox at the range of 0.03 to 2 μg/mL induced dramatic cell number loss, and reduced the IC_50_ of Dox from 0.8 μg/ml to 0.08 μg/ml (Fig. 2d). We calculated the isobologram index (Ix) of Dox and LBH589 as 0.41 according to the equation in Material and Methods. Thus, KLF9 expression and LBH589 acted synergistically to induce GSC number loss. A similar pattern of synergistic cell number loss induced by KLF9 expression and LBH589 was observed in GBM1B cells (data not shown).

### LBH589 affected cell cycle but mitotic catastrophe was not involved in cell death induced by KLF9 expression + LHB589

**Fig. 3:**
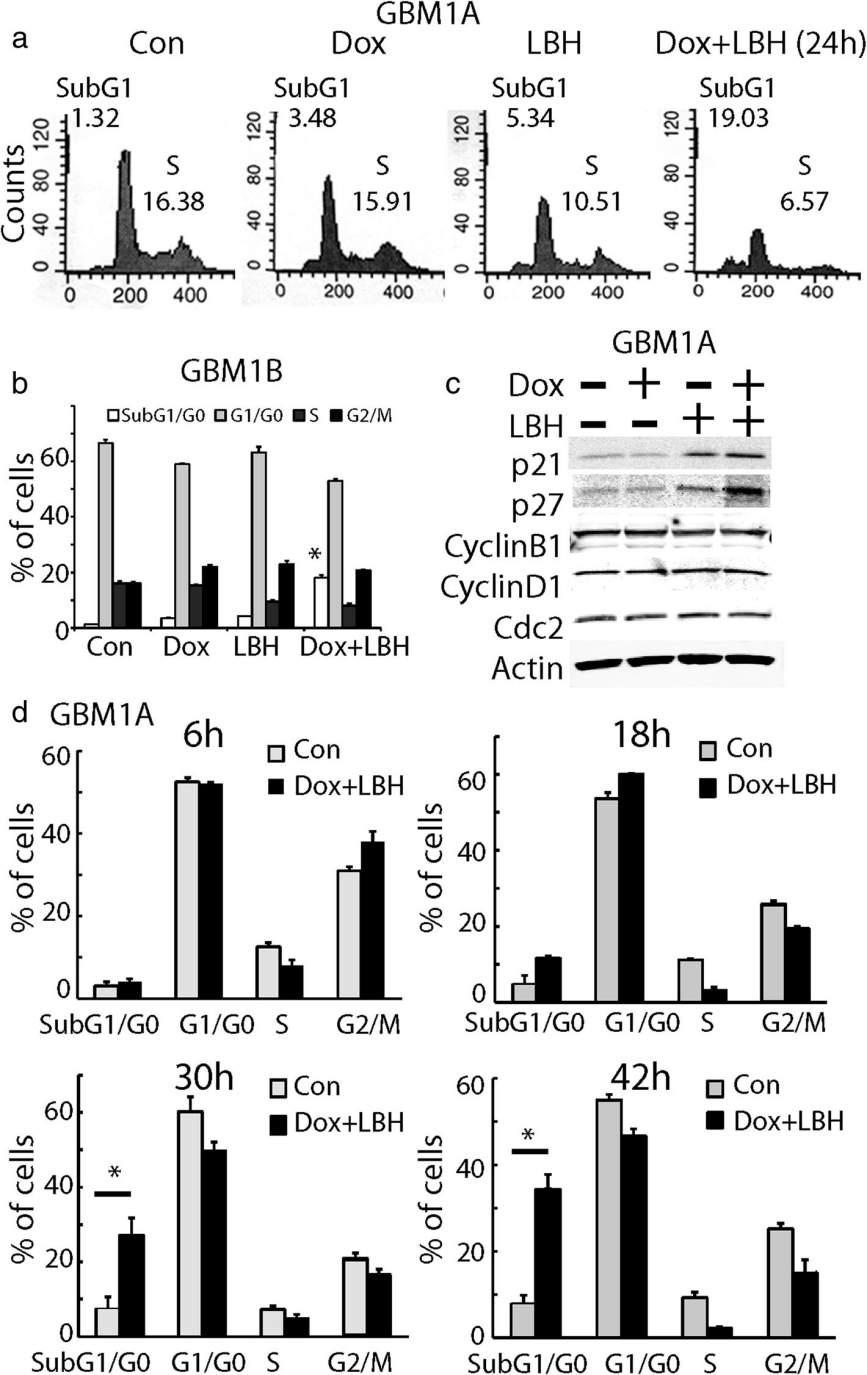
HDAC inhibitor LBH589 induced cell cycle arrest but not mitotic catastrophe. **a** Cell cycle analysis of GBM1A cells treated with Dox, LBH589, and Dox + LBH589 for 24 h. Cells were not synchronized before experiments. In the combined treatment of LBH589 and Dox, cell cycle was arrested at S-phase (6.57%) and there was a significant increase in sub-G1/G0 portion (~ 19%), indicating cell death. **b** Similar cell cycle experiment results were found in GBM1B neurosphere cells. **c** Expression of cell cycle modulators including cyclins, p21, p27 and cdc2 were examined in GBM1A cells treated with Dox and LBH589 alone or combined. **d** Time course of cell cyle progression under the treatment of Dox + LBH589. Cells were growth arrested by thymidine for 8 h followed by release from cell cycle arrest for indicated time points. Cell cycle analysis indicated that at 18 h, Dox + LBH589-treated cells had a slight decrease at G2/M phase without increase in cell accumulation at sub-G1/G0 phase. At 30 h and 42 h, there was a dramatic decrease in cells at G2/M phase and a slight increase in cells at sub G1/G0 phase, indicating no accumulation of cells in G2/M phase before cell death. *: *P* < 0.05

HDAC inhibitors have been reported to induce cell cycle arrest [39]. To examine the effect of the combined treatment on cell proliferation, we examined cell cycle progression in GSCs under different treatments by flow cytometry analysis of propidium iodide (PI) stained cells. We first analyzed cell cycle without synchronization. After 24 h of treatment, LBH589 alone decreased the percentage of cells at S-phase from 16.38 to 10.51%, and there was a slight increase in cells at sub-G0/G1 phase from 1.32 to 5.34% (Fig. 3a). In parallel, we found that Dox alone did not induce cell growth arrest, yet Dox + LBH589 further decreased the percentage of cells at S-phase, from 16.38 to 6.57%, and significantly increased the percentage of cells at sub-G1/G0 phase (from 1.32 to 19.03%, *P* < 0.05) (Fig. 3a). This was consistent with our previous data showing that enhanced cell death was induced by the combined treatment. Similar results were found in GBM1B cells (Fig. 3b). We examined the expression of cell cycle regulators influenced by Dox and LBH589. LBH589 alone or in combination with Dox upregulated the expression of cell cycle inhibitors p21 and p27. The expression of cyclin B, D1 and other cell cycle regulators such as cdc2 were not changed by LBH589 or Dox (Fig. 3c).

It has been shown that cells with a dysregulated cell cycle could mistakenly enter into mitotic phase and eventually die via a process called mitotic catastrophe in which cells die via both apoptosis and necrotic death [40]. Since mitotic catastrophe has been reported in HDAC-treated GBM cells [41], and our cell cycle analysis indicated that Dox + LBH589 dramatically inhibited cell cycle progression at S-phase, we tested whether the enhanced cell death in KLF9 expressing cells by LBH589 might occur via mitotic catastrophe. A hallmark of mitotic catastrophe is an accumulation of cells at G2/M which then die abruptly [40]. We synchronized the cells with thymidine and then treated the cells with Dox + LBH589. Cell cycle progression was analyzed during the time course of drug treatment at 6 h, 18 h, 30 h and 42 h (Fig. 3d**)**. Six hours after GSCs were released from the cell cycle blocker, the control cells progressed from S (12.3%) to G2/M phase (31.2%). In the Dox + LBH589 treated cultures, there was no cell death after 6 h, cells at S-phase was 6.7%, and there was a slight increase in cells at G2/M phase (38.9%). After 18 h of treatment, the Dox + LBH589 treated cells had fewer cells in S-phase (3.8%), and decreased cells at G2/M phase compared to control (19.5% vs 23.5%). Moreover, there was an increase in cells at sub-G1/G0 phase, from 4.87 to 11.5% in the control and Dox + LBH589 treated group, respectively. After 30 h and 42 h of the combined treatment, there was a substantial increase in cells at sub-G1/G0 phase (28–35%). Yet, we did not observe an increase in cells at G2/M phase in the cells prior to the significant increase in cell death (at 18 h). Our data indicated that cell death induced by the combined treatment did not occur via mitotic catastrophe, which is characterized by G2/M accumulation.

### KLF9 and LBH589 regulated the expression of pro- and anti- apoptotic proteins

**Fig. 4:**
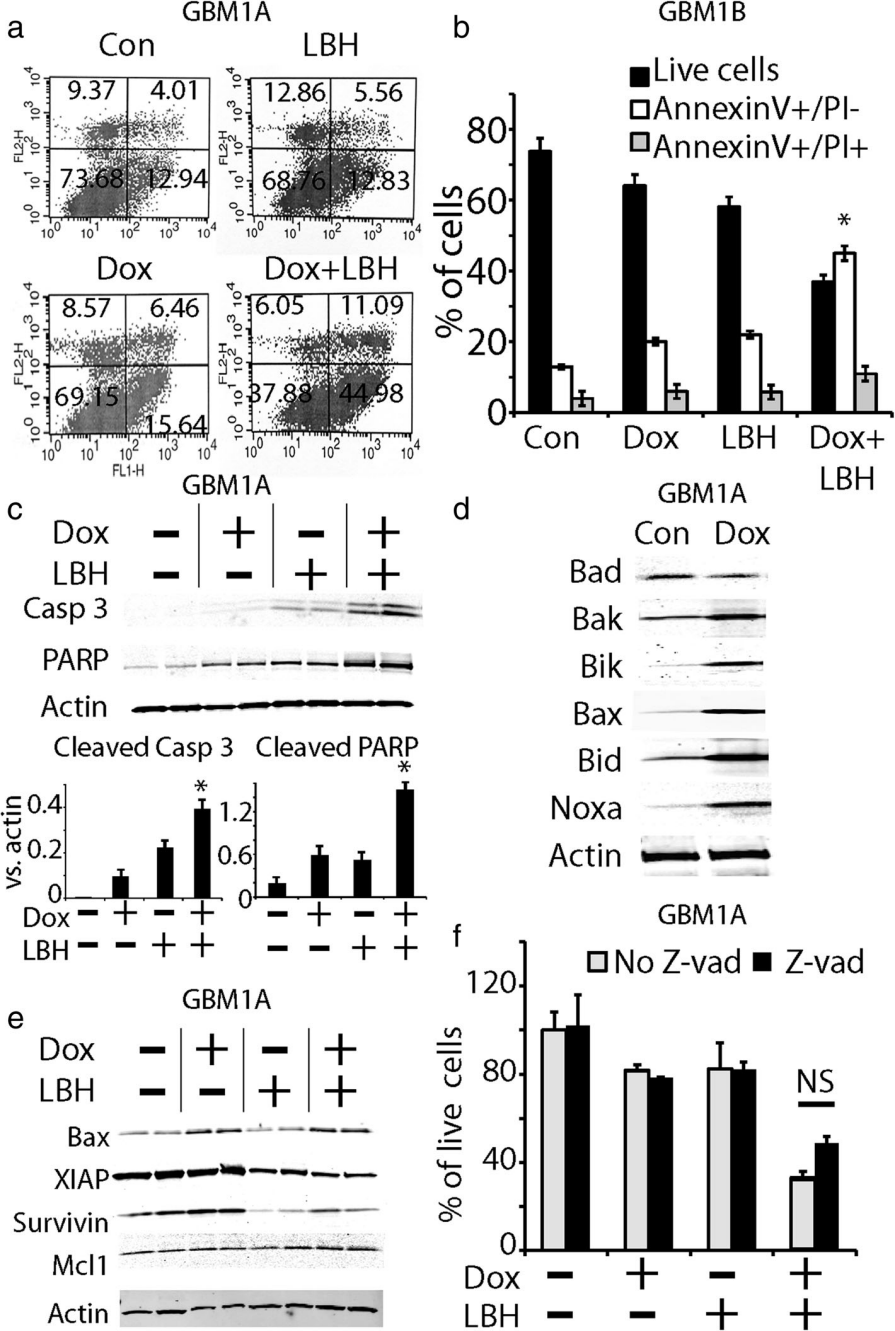
Forced KLF9 expression and HDAC inhibitor LBH589 induced apoptosis. **a**, **b** Early stage apoptosis (Annexin-V positive, PI negative) and late stage apoptosis or necrosis (Annexin-V positive, PI positive) in GBM1A cells (A) under different treatment for 24 h were measured by flow cytometry. Similar experiments were performed in GBM1B cells (B)**. c** Increased cleaved caspase 3 and cleaved PARP were detected in GSCs treated with Dox + LBH589. **d** Expression of pro-apoptotic proteins was examined in GSCs. Pro-apoptotic proteins Bak, Bik, Bax, Bid and Noxa were up-regulated by KLF9 expression. **e** Expression of anti-apoptotic proteins survivin and XIAP was down regulated by LBH589 alone or in the presence of Dox. **f** The pan-caspase inhibitor z-vad had minimal protective effect on KLF9-expressing GSCs treated with LBH589. *: *P* < 0.05

The morphology of dead cells suggests that concomitant KLF9 expression and LBH589 induced a mixture of apoptosis and necrosis (Fig. 1f). We further characterized the cell death by flow cytometry analysis with annexin V–FITC/PI staining, which quantifies both apoptotic and necrotic cell death. Annexin V+ /PI- cells indicate early stage of apoptosis, whereas annexin V+/PI + cells include both late stage apoptosis and necrosis. After 48 h of treatment, the early stage apoptotic portion of cell death (annexin-V+/PI-) was found to be 12.94% in GBM1A control cells, 12.83% with LBH589 alone, 15.64% with Dox alone, and 44.98% with Dox + LBH589 (Fig. 4a). Furthermore, the late-stage apoptotic and necrotic cell death (annexin-V+/PI+) were found to be 4.01% with control, 5.56% with Dox alone, 6.46% with LBH589 alone, and 11.09% with Dox + LBH589 (Fig. 4a, b). Thus, Dox or LBH589 alone did not induce cell death (apoptosis and necrosis). Dox + LBH589 induced more than 3-fold increase in the early stage apoptosis (from 12.94 to 44.98%, *P* < 0.001) and 2.6-fold increase in the late stage of apoptosis or necrosis (from 4.01 to 11.09%). Our flow cytometry data suggests that the enhanced cell death by KLF9 expression + LBH589 was largely due to apoptosis.

Next, we determined the mechanism of apoptotic cell death induced by KLF9 expression and LBH589. We first examined whether KLF9 expression + LBH589 induced caspase 3 and PARP activation. In cells treated with Dox + LBH589, there was an increase in caspase 3 and PARP cleavage as illustrated and quantified by Western blotting analysis (Fig. 4c), indicating involvement of caspase-dependent apoptotic pathways in the synergistic cell death.

To identify the gene targets underlying the enhanced apoptosis in GSCs, we examined the expression of pro-apoptotic and anti-apoptotic proteins induced by KLF9 and LBH589. KLF9 upregulated the expression of pro-apoptotic proteins including Bak, Bik, Bax, Bid and Noxa (Fig. 4d). The expression of Bad was not changed in our cell models. The combination of KLF9 induction and LBH589 increased the expression of the apoptotic protein Bax to the comparable level of that with Dox alone (Fig. 4e). LBH589 alone dramatically reduced the expression of anti-apoptotic proteins, XIAP and survivin (Fig. 4e), whereas the expression of other anti-apoptotic proteins such as MCL1, were not changed by LBH589 (Fig. 4e). Further, Dox alone did not change the expression level of XIAP and survivin. KLF9 expression + LBH589 decreased the expression level of anti-apoptotic proteins, XIAP and survivin (Fig. 4e). Thus, KLF9 + LBH589 upregulated pro-apoptotic proteins and downregulated anti-apoptotic proteins, ultimately inducing cells to undergo apoptosis.

Although we have demonstrated that the combination of KLF9 and LBH589 activated increased expression of caspase 3, and the ratio of pro- and anti-apoptotic proteins were changed to favor apoptosis, to our surprise, the pan-caspase inhibitor, z-vad-fmk (z-vad), provided a limited protective effect against cell death induced by KLF9 expression + LBH589. After 48 h of incubation, Dox + LBH589 induced 81% cell loss as measured by MTS. However, in the presence of z-vad, Dox + LBH589 still induced 68% cell loss (Fig. 4f). In other words, z-vad only marginally rescued cells from cell death induced by the combined treatment, compared with ~ 45% of early stage apoptosis measured by flow cytometry (Fig. 4a).

### KLF9 and LBH589 induced programmed necrosis (necroptosis) in GSCs

**Fig. 5:**
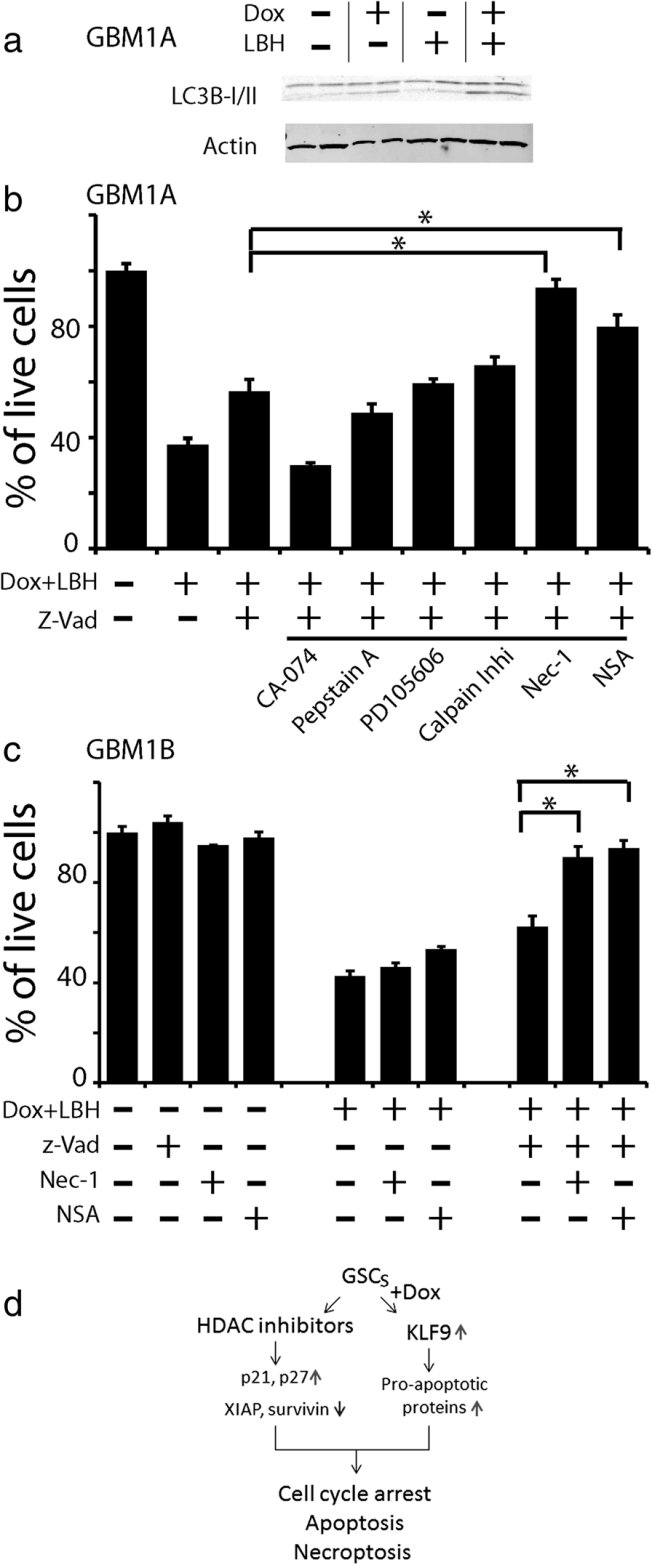
Forced KLF9 expression and HDAC inhibitor LBH589 induced necroptosis in GSCs. **a** Western blot analysis indicated that there was no activation of autophagy marker LC3B-I in GSC cultures under different treatment conditions. **b** The combination of z-vad with calpain inhibitors or calpaithin inhibitors had minimal effect to protect KLF9-expressing GBM1A cells from death induced by LBH589. Two necroptosis inhibitors, Nec-1 and NSA, together with z-vad, significantly rescued GBM1A cells from death induced by Dox + LBH589. **c** Nec-1 or NSA alone did not protect GBM1B cells from death induced by Dox + LBH589. Cell death was completely blocked only when both apoptosis inhibitor and necroptosis inhibitor were applied together in GBM1B cells treated with Dox + LBH589. *: *P* < 0.05. **d** A model of the synergistic anti-tumor effect by combining HDAC inhibitors and KLF9 expression in GSCs

Our observation that pan-caspase inhibitor z-vad had minimal protective effect against GSC death induced by Dox + LBH589 suggests that KLF9 induction and HDAC inhibitor LBH589 may promote caspase-independent cell death. We sought to identify if autophagy was involved in the enhanced cell death induced by Dox + LBH589. No activation of autophagy marker LC3B-I was detected in cultures under different treatment conditions, alone or combined. Only a slight increase in LC3B-II was detected in Dox and combined treatment groups, indicating that autophagy was not responsible for the substantial cell death induced by the combined treatment (Fig. 5a).

Besides apoptosis, we also examined necrosis in GSCs under drug treatments. Necrotic cell death could occur via a calpain or cathepsin-mediated mechanism. We quantified cell death in the presence of specific necrosis inhibitors including calpain and cathepsin inhibitors. Compared with z-vad alone, the calpain and cathepsin inhibitors together with z-vad did not significantly protect against cell death induced by Dox + LBH589 in GBM1A cells (Fig. 5b).

Programmed necrosis, also known as necroptosis, represents necrosis controlled by defined cellular death pathways [42]. Of note, necroptosis can be induced by z-vad [43]. Based on the observation that apoptosis was the major form of cell death induced by KLF9 expression and LBH598, yet z-vad failed to elicit protective effect in GSCs, we hypothesized that necroptosis might explain the lack of protection by z-vad. We therefore quantified cell death in the presence of z-vad and specific necroptosis inhibitors necrostatin-1 (Nec-1) [44] and necrosulfonamide (NSA) [45]. The combination of Nec-1 with z-vad significantly blocked cell death induced by Dox + LBH589 in GBM1A and GBM1B cells (Fig. 5b, c). Similar results were observed when the cells were treated with NSA (Fig. 5b, c). Finally, we investigated whether necroptosis was involved in cell death induced by KLF9 expression and LBH598. Nec-1 and NSA possessed no protective effect when applied alone in the Dox + LBH589 treated GSC cultures (Fig. 5c). All these data suggest the involvement of necroptosis in KLF9 expressing GSCs when treated together with HDAC inhibitors and the apoptosis inhibitor z-vad.

## Discussion

In the present work, we found that combining two differentiation regimens, namely the transcription factor KLF9 and HDAC inhibitors, triggered synergistic cell death in GSCs via a mechanism involving both apoptosis and necroptosis (Fig. 5d). Given the resistance of GSCs to conventional radiation and chemotherapy, the tumor-cell killing effect of combined differentiation therapies will likely open a new opportunity to treat these refractory GSCs.

The tet-on system with Dox-inducible KLF9 expression was used in this study as it provided an internal controlled model for KLF9 functional analysis [9, 17]. In other words, if we had investigated KLF9 function with stable cell lines that constitutively express KLF9, we would have compared cellular responses to HDAC inhibitors in two different cell lines, whose behavior may have differed during the long process of establishing cell lines from single clones, thus making the interpretation of the results confounded. In our study, we used several methods to quantify cell number and cell death including MTS assays, trypan blue staining, annexin-V/PI staining, and cell cycle analysis. All these assays demonstrated enhanced cell death induced by KLF9 expression + LBH589. Because of the different quantification methods, we acknowledge that there may be some variation on the percent of cell death induced by each agent alone or combined. In part, this may be because some methods measure both cell death and proliferation while other methods exclusively quantify cell death. For example, MTS assays measure both cell proliferation and cell death, whereas trypan blue and annexin-v staining measure the percentage of dead cells under treatment conditions. In addition, we used cell cycle analysis to measure percentage of cells at the sub G1/G0 phase as an indicator for cell death. We noticed that even though LBH589 treatment alone induced significant cell cycle arrest, these was no increase in the sub G1/G0 portion, confirming LBH589’s effect on cell proliferation without inducing cell death. The delayed S-phase may be due to increased stem cell differentiation under LBH589 treatment, as previously reported by our group [7]. In our previous studies, we found that HDAC inhibitors TSA and MS-275 induced differentiation of GSCs with no effect on cell death [7]. This is consistent with our current work that LBH589 induced cell cycle arrest in GSCs as evidenced by cell cycle analysis and increase expression in p21 and p27 on Western blot analysis.

Different types of cell death have been investigated in our study: we characterized apoptosis, autophagy, mitotic catastrophe, and necroptosis following the treatment of LBH589 in KLF9 expressing cells. We found that the enhanced cell death induced by HDAC inhibitors and forced KLF9 expression was a mixture of apoptosis and necroptosis. Several scenarios may explain the synergistic cell death in the context of dual KLF9 expression and LBH589 administration. First, compared to undifferentiated stem-like cells that are more resistant to chemotherapeutic drugs, differentiated cells induced by KLF9 expression may be more vulnerable to anti-cancer drugs, such as HDAC inhibitors. Second, our previous RNA-seq and ChIP-seq data indicated that KLF9 regulated the gene expression of both pro-apoptotic and anti-apoptotic proteins [17]. This was confirmed in our current study that KLF9 dramatically upregulated pro-apoptotic proteins including Bak, Bik, Bax, Bid and Noxa. We also examined the expression of a panel of apoptosis regulators in KLF9 expressing cells in the presence of LBH589, and found that there was a dramatic downregulation of anti-apoptotic proteins XIAP and survivin. The synergistic effect of KLF9 expression and LBH589 elicits an enhanced cell death response in GSCs. When we examined our KLF9 ChIP-seq gene list, we found that KLF9 directly binds to the promoter regions of Bak, Bik, Bax, Bid and Noxa, [17] but not to the promoter regions of XIAP and survivin. Therefore, we conclude that in the presence of decreased anti-apoptotic proteins, KLF9 directly upregulated pro-apoptotic gene expression to enhance cell death.

Perhaps the most interesting finding is the involvement of programmed necrosis (necroptosis) induced by KLF9 expression + LBH589 in the presence of apoptosis inhibitor z-vad. The fact that pan-caspase inhibitor z-vad did not proportionally protect GSC death induced by Dox + LBH589 suggests that [1] Dox + LBH589 induced caspase-independent cell death, and/or [2] Dox + LBH589 + z-vad induced a new form of cell death. The combination of z-vad with necroptosis inhibitors significantly rescued GSC death, suggesting that apoptotis and necroptosis may simultaneously occur in KLF9 expressing cells when treated with LBH589. Upon examining the expression of the necroptosis effector receptor-interacting protein (RIP) [46], we found no significant change in RIP1/3 following drug treatment (data not shown). The exact mechanism and regulatory network by which KLF9 and HDAC inhibitors regulate the expression of necroptotic effectors and activate necroptosis in our system is currently unknown. Further analyses in the future will help to identify novel targets for anti-tumor treatments.

Moreover, the manipulation of nuclear proteins for cancer treatment is an exciting area of research. Compared with small molecular inhibitors that target receptors and/or kinases on cell membranes or in the cytoplasm that inevitably generate escape mechanisms, employing transcription factors such as KLF9 to target cancer stem cells would be beneficial because these proteins tightly control gene expression upstream of signaling transduction pathways, thereby preventing cells from developing compensatory mechanisms. With advanced gene therapy technology, our prospective in vivo testing of the synergistic cell death via KLF9 and HDAC inhibitors will help in developing new anti-tumor strategies for GBM patients.

## Conclusions

Tumors are heterogeneous and comprised of a small group of tumor-initiating or cancer stem cells (CSCs). CSCs are resistant to current chemotherapy and radiotherapy, leading to metastasis and relapse of cancers, therefore significantly affect cancer therapy. In this study, we investigated the combined treatment of epigenetic modulators and forced expression of the transcription in inducing cell death in glioblastoma stem cells (GSC). We found that the combination of histone deacetylase (HDAC) inhibitors and expression of krÜppel-like factor 9 (KLF9) synergistically promote GSC death through a mechanism involving both apoptosis and necroptosis. Our findings are expected to benefit the development of effective anti-tumor strategies to treat malignant brain tumors.
